# Supplementary material for: Boundary sequences flanking the mouse tyrosinase locus ensure faithful pattern of gene expression
Source: Sci Rep. 2020 Sep 23;10:15494. doi: 10.1038/s41598-020-72543-0 (PMC7511308; doi:10.1038/s41598-020-72543-0)
Supplement: Supplementary file 1 — Supplementary Information [file 41598_2020_72543_MOESM1_ESM.pdf]

Boundary sequences flanking the mouse tyrosinase locus ensure faithful pattern of gene expression

Davide Seruggia[1,2], Almudena Fernández[1], Marta Cantero[1], Ana Fernández-Miñán[3], José Luis Gomez-Skarmeta[3], Pawel Pelczar[4] and Lluis Montoliu[1,*]

[1]Department of Molecular and Cellular Biology, National Centre for Biotechnology (CNB-CSIC) Madrid, Spain and CIBERER-ISCIII, Madrid, Spain

[2] Current address: Division of Hematology/Oncology, Boston Children's Hospital, Harvard Medical School, Boston, MA, USA

[3]Centro Andaluz de Biología del Desarrollo (CABD), CSIC-Universidad Pablo de Olavide-Junta de Andalucía, Seville, Spain;

[4]Center for Transgenic Models, University of Basel, Basel, Switzerland

* Address for correspondence:

Lluís Montoliu

Centro Nacional de Biotecnología (CNB-CSIC)

Campus de Cantoblanco

Darwin 3

28049 Madrid, Spain

Tel +34 915854844

Fax. +34 915854506

Email: montoliu@cnb.csic.es

SUPPLEMENTARY TABLES AND FIGURES

SUPPLEMENTARY TABLE 1: Oligonucleotide sequences

**Primers used for 3C**	
**Anchor on *Tyr* promoter**	
AGGGGTTGCTGGAAAAGAAG	Tyr_promoter
TTTTCTTGACTTTGTGTCCCTATG	Tyr1
TCTTAATGTTGGAAAGTGCAAAGT	Tyr2
TCACAACACTGTCATACCATCTG	Tyr3
TTGGACCTGTGCTGTGACTAA	Tyr5
AAGGACGGAGTGGAGGTTG	Tyr6
GGCAGGATGGGACTGAAGTA	Tyr11
ATTTGTCTGGGGGCTCATAAC	Tyr12
CACACAACTATCACTACTATCACCAC	Tyr13
TGTTGAATCCCACCTTTACTCC	Tyr14
CCATGCCCTGCTAAATGTGTA	Tyr15
CCCAGACCCTTCCAAGTCAGTAT	Tyr16
TGGAAAATGAGACACAACGAAG	Tyr17
**Anchor on Tyr 5’**	
CACACAACTATCACTACTATCACCAC	Tyr_5'
TCACAACACTGTCATACCATCTG	Tyr3
AAAGACACCATCCCTCCAAC	Tyr7
TTCTCTTTTCTTTCGCACCA	Tyr8
AGGGGTTGCTGGAAAAGAAG	Tyr9
CCCAGACCCTTCCAAGTCAGTAT	Tyr16
TACAGCAACACATTAGAACCAGA	Tyr17
**Anchor on CNS-2**	
CCCAGACCCTTCCAAGTCAGTAT	Tyr_CNS2
TCTCAAATCCCTCCTATCCAA	Tyr4
TTGGACCTGTGCTGTGACTAA	Tyr5
AAGGACGGAGTGGAGGTTG	Tyr6
AGGCTGAGAGTATTTGATGTAAGAA	Tyr10
CACACAACTATCACTACTATCACCAC	Tyr13
TACAGCAACACATTAGAACCAGA	Tyr17
***Ercc3* locus**	
GTCTGTCTTTGTTGCTGAAGTATG	XBP1
AAGTCCAGTGTGCTGAGGTATT	XBP2
**Primers used for cloning EBA vectors**	
CAGCTCGAGACAGAAATGGCCCCACCTAT	XhoI_tyr3'F (2.5kb)

CAGCTCGAGTGCATTTGAACTTGACCTACTGA	XhoI_tyr3'R (2.5kb)
CAGCTGCAGACAGAAATGGCCCCACCTAT	PstI_tyr3'F (2.5kb)
CAGCTGCAGTGCATTTGAACTTGACCTACTGA	PstI_tyr3'R (2.5kb)
CAGCTGCAGCCAGGTGAGGGGTGTGTTTA	XhoI_tyr3'F (241bp)
CAGCTGCAGGAAGTGTTTATTGACAATGTG	XhoI_tyr3'R (241bp)
CAGCTCGAGCCAGGTGAGGGGTGTGTTTA	PstI_tyr3'F (241bp)
CAGCTCGAGGAAGTGTTTATTGACAATGTG	PstI_tyr3'R (241bp)
TGTCTTCAGACACTCGAGAATAGAGCGCCAGATCTTGTTA	3'CTCFmutF
TAACAAGATCTGGCGCTCTATTCTCGAGTGTCTGAAGACA	3'CTCFmutR
**primers for cloning of sgRNAs**	
ACACCAGCTCAGTAGAGTACTAGGTG	Tyr3'3gRNAFw
AAAACACCTAGTACTCTACTGAGCTG	Tyr3'3gRNARv
ACACCACAAACGCTAATTGGTAAAAG	Tyr3'5gRNAFw
AAAACTTTTACCAATTAGCGTTTGTG	Tyr3'5gRNARv
**Primers for mouse genotyping**	
CAACCAGGCTTTCATCAGAAT	Tyr5_delF
TTTTCTCTGTATCATGATTGCCTA	Tyr5_delR
TCTGTGCATGGTATACAACAGGG	Tyr3_delF
GTGCATTAAAGGAAGCCCAATGA	Tyr3_delR

Supplemental Figure 1

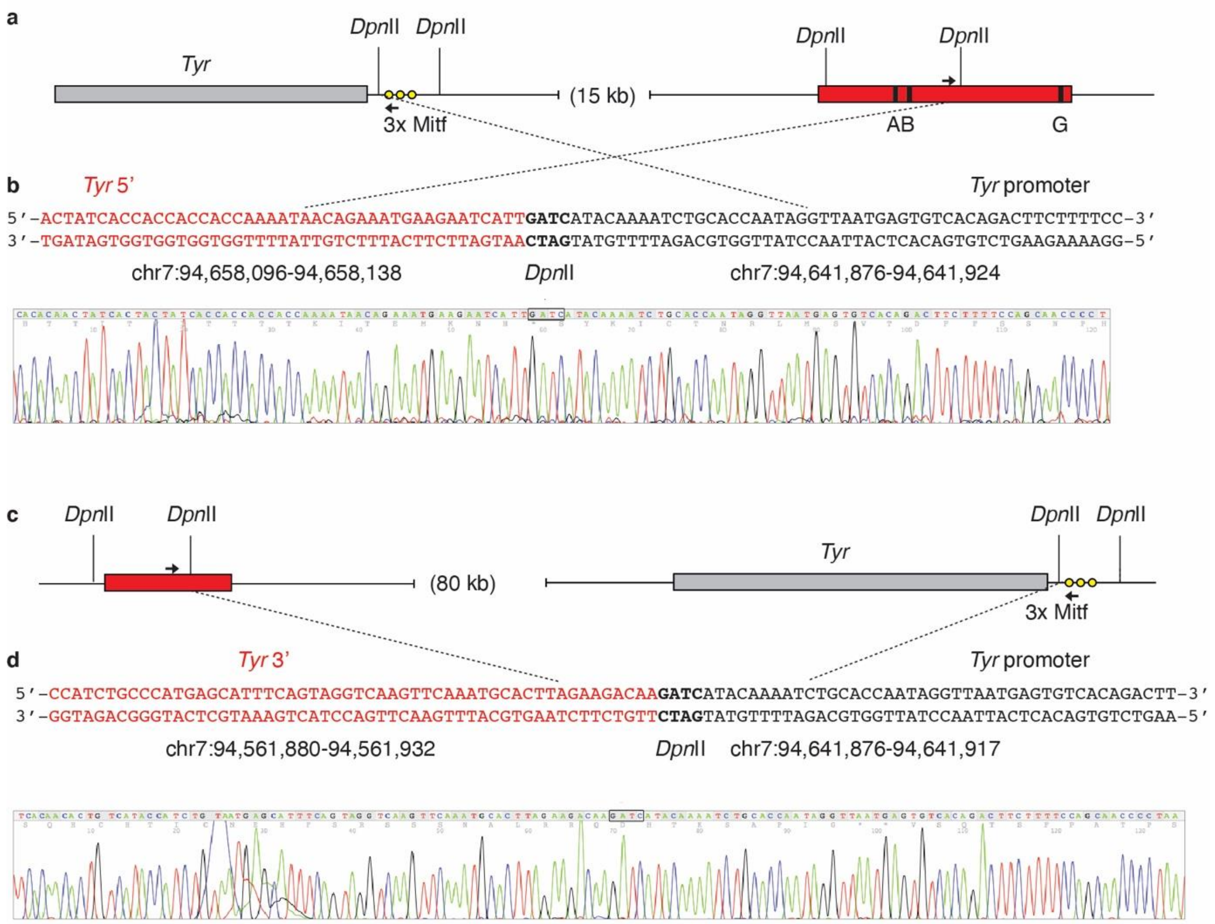

Supplemental Figure 2

a

chr7-94,656,561-94,656,970 (mm9); **Sox10**, **Mitf**

ATAACAGGAAAATAAACAAGCCACATTCCTGCTTTGTAGAAGTTCACAATCTGAATAGGAATATAAAAAGGTGATGGTGTGGCAATTTCAGGACTCTTTATTGGGTACA

TATTGTTAATAATATTGTGGTTTGCCAGGACCCCAGCAGGAAGCAGCTGACACACTCAAGCTGGTTCTTTGAACAGAGTTT**AAGAAAGAAGCTATTTA**CAATGTTGGGG

B box — A box

GTGGAATTTA**AGAAGAAAGGACAGTGGA**TAATGAAAAGGATACTCAGAACAACTGCT**GGAACACAGACATCACTGTC**TTGACAA**AGTCAGCTGGC**AGGAACTATGACCT

USF-1 motif

TGGAGAAGGATATAAGCAACTTTCTTGTTATGTGGCAAAGAGGAAGTTGCAGACCTTTGACTTCACTCTCCTTCTGCCTCTAA

b

TRANSFAC

TF	KO mouse	pigmentation
Ets	yes	normal
Cdx2	lethal	n.a.
Runx2	yes	normal
Sry	yes	normal
Nfatc2	yes	normal
Nr5a2	lethal	n.a
Myb	yes	normal
Ttf1	n.a.	n.a
Bptf	lethal	n.a.
**Lef1**	**yes**	**aberrant melanocytes with no melanin**

UNIPROBE

TF	KO mouse	pigmentation
Lmx1b	yes	normal
Sry	yes	normal
Pou4f3	yes	normal
**Lmx1a**	**yes**	**white spotted**
Arid1a	yes	normal
Sox7	lethal	n.a.
Sox12	yes	normal

Supplemental Figure 3

a

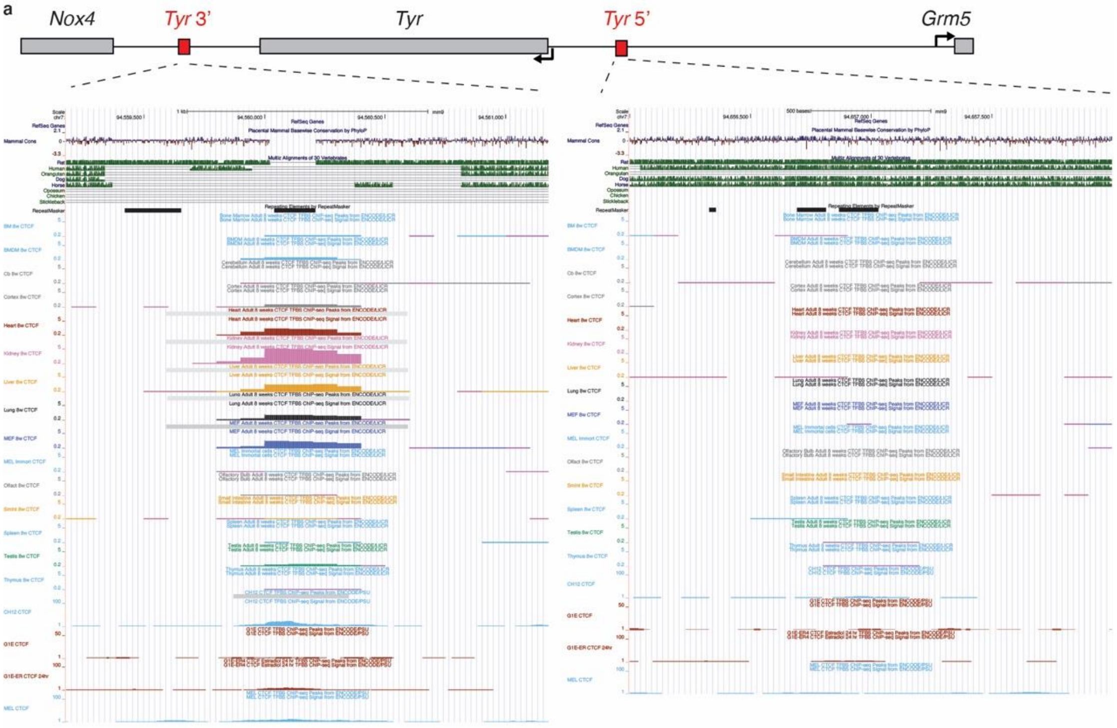

b

GAAGTGTTTATTGACAATGTGACATTTTGTTTTG**TTTTGTTTTTTAAAGATTTATTTATTTATTATATGTAAGTTC**
ACTGTAGCTGTCTTCAGACACTCCAGAAGAGGGCGCCAGATCTTGTTACGGATGGTTGTGAGCCCCCATGTGGTTG
ACCTTCGGAAGAGCAGTCAGTGCTCTTAGCTGCTGAGCCATCTCTCCAGCCCTGACATTGTGATATTTTTAAACAC
ACCCCTCACCTGG

chr7:94560004-94560244 (minus strand)

c

GAAGTGTTTATTGACAATGTGACATTTTGTTTTGTTTTGTTTTTTAAAGATTTATTTATTTATTATATGTAAGTTC
ACTGTAGCTGTCTTCAGAC**ACTCCAGAAGAGGGCGCCAG**ATCTTGTTACGGATGGTTGTGAGCCCCCATGTGGTTG
ACCTTCGGAAGAGCAGTCAGTGCTCTTAGCTGCTGAGCCATCTCTCCAGCCCTGACATTGTGATATTTTTAAACAC
ACCCCTCACCTGG

d

5′-ACTCCAGAAGAGGGCGCCAG-3′ wt

5′-ACTC**G**AGAA**T**AG**A**GCGCCAG-3′ mutated

Supplemental Figure 4

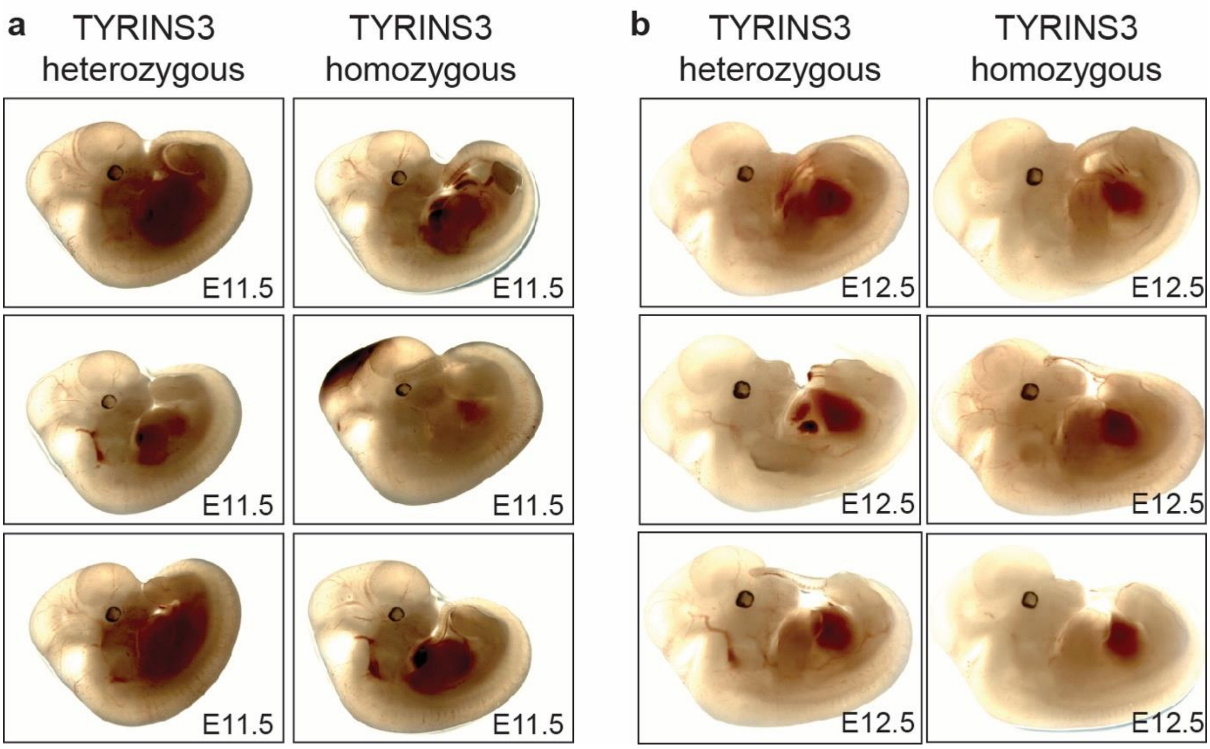

SUPPLEMENTARY FIGURE LEGENDS

Supplemental Figure S1: Chromosome conformation of the mouse *Tyr* locus.

(**a**) Diagram of the DpnII restriction sites at the *Tyr* promoter and *Tyr* 5' element. MITF binding sites at the *Tyr* promoter are indicated as yellow circles. A, B and G boxes are highlighted in black. 3C primers are depicted as black arrows (**b**) Sanger sequencing of chimeric ligation products resulting by proximity ligation of DpnII fragments at the *Tyr* promoter and *Tyr* 5' element. (**c**) Diagram of the DpnII restriction sites at the *Tyr* promoter and *Tyr* 3' element. (**d**) Sanger sequencing of chimeric ligation products resulting by proximity ligation of DpnII fragments at the *Tyr* 3' element and *Tyr* promoter. Genomic coordinates are indicated (mm9). Figure prepared with Adobe Illustrator.

Supplemental Figure S2: The *Tyr* 5' element contains a melanocyte-specific *Tyr* enhancer

(**a**) Sequence of the *Tyr* 5' core enhancer. Sox10 binding motifs are highlighted in red; Mitf motif is highlighted in green. A and B box sequences are boxed; the Usf-1 motif, carrying insulator activity, is indicated (**b**) Additional transcription factor binding motif predictions using TRANSFAC and UNIPROBE. Figure prepared with Adobe Illustrator.

Supplemental Figure S3: The *Tyr* locus is flanked by chromatin boundaries

(**a**) Genomic view of the *Tyr* 3' and *Tyr* 5' elements. Mammalian sequence conservation and repeat DNA tracks from USCS Genome Browser. CTCF occupancy in mouse cell lines and tissues from ENCODE. (**c**) Sequence of the *Tyr* 3' core element; the SINEB2 sequence is highlighted in red. The CTCF binding motif is highlighted in red. (**d**) CTCF binding motif compared with its mutagenized version used in zebrafish. Figure prepared with the help of Adobe Illustrator program.

Supplemental Figure S4: Deleting the *Tyr* 3' element in mice does not alter the pattern *Tyr* gene expression. (**a**) Heterozygous and homozygous TYRINS3 E11.5 embryos (**b**) Heterozygous and homozygous TYRINS3 E12.5 embryos. Figure prepared with Adobe Illustrator. The images included in this figure have been obtained by Davide Seruggia at the CNB-CSIC in Madrid under the supervision of Lluis Montoliu.
